# Test, evidence, transition projects in Scotland: developing the evidence needed for transition of effective interventions in cancer care from innovation into mainstream practice

**DOI:** 10.1186/s12885-023-11592-w

**Published:** 2023-11-01

**Authors:** Erica Wirrmann Gadsby, Carson Brown, Claire Crawford, Glen Dale, Edward Duncan, Linda Galbraith, Karen Gold, Carina Hibberd, Agi McFarland, Jennifer McGlashan, Melanie McInnes, Joanne McNaughton, Juliette Murray, Esme Radin, Piotr Teodorowski, Jane Thomson

**Affiliations:** 1 Faculty of Health Sciences and Sport, University of Stirling, Pathfoot Building, Stirling, FK9 4LA UK; 2 NHS Forth Valley, 33 Spittal Street, Stirling, FK8 1DX UK; 3 NHS Fife, Victoria Hospital, Hayfield Road, Kirkcaldy, KY2 5AH UK; 4 Patient/public representative, University of Stirling, Stirling, FK9 4LA UK

**Keywords:** Cancer diagnosis, Implementation science, Scotland, Evaluation, Prostate cancer, Breast cancer

## Abstract

**Background:**

A robust evidence base is required to assist healthcare commissioners and providers in selecting effective and sustainable approaches to improve cancer diagnosis and treatment. Such evidence can be difficult to build, given the fast-paced and highly pressured nature of healthcare delivery, the absence of incentives, and the presence of barriers in conducting pragmatic yet robust research evaluations. Cancer Research UK (CRUK) has played an active part in building the evidence base through its funding of programmes to identify, evaluate and scale-up innovative approaches across the UK. The aim of this paper is to describe and explain the research design and intended approach and activities for two cancer services improvement projects in Scotland funded by CRUK.

**Methods:**

A hybrid effectiveness-implementation study design will assess both the efficiency of the new pathways and their implementation strategies, with the aim of generating knowledge for scale-up. A range of implementation, service and clinical outcomes will be assessed as determined by the projects’ Theories of Change (ToCs). A naturalistic case study approach will enable in-depth exploration of context and process, and the collection and synthesis of data from multiple sources including routine datasets, patient and staff surveys, in-depth interviews and observational and other data. The evaluations are informed throughout by a patient/public representatives’ group, and by small group discussions with volunteer cancer patients.

**Discussion:**

Our approach has been designed to provide a holistic understanding of how (well) the improvement projects work (in relation to their anticipated outcomes), and how they interact with their wider contexts. The evaluations will help identify barriers, facilitators, and unanticipated consequences that can impact scalability, sustainability and spread. By opting for a pragmatic, participatory evaluation research design, we hope to inform strategies for scaling up successful innovations while addressing challenges in a targeted manner.

## Background

In the organisation and delivery of cancer care in the United Kingdom, the importance of meeting patient needs and achieving government targets is set against the constraints of limited resources. Intervals between referral for suspicion of cancer, confirmation of diagnosis and beginning of treatment are all used by the UK government (and devolved governments) as indicators of quality in cancer care. Reducing such intervals to a minimum is intended to limit the stress and anxiety for people as well as catch cancer at a point where treatment is more likely to be successful. However, adherence to these targets (particularly for the 62-day wait from urgent General Practice (GP) referral to first treatment) is historically poor and highly varied by region and cancer type [1–4]. Any nation committed to providing equitable, responsive and high-quality healthcare services, faces considerable challenges arising from the growing demand for comprehensive cancer care, as cancer cases increase in number and complexity [5, 6]. To deliver timely and effective services, while grappling with the realities of constrained budgets and workforce shortages requires innovative solutions [7, 8].

Healthcare providers are excellent innovators, frequently looking for new ways of working, often demonstrating creativity in devising strategies that streamline processes, optimise resource allocation, and enhance the overall quality of care. But many of these innovations are neither documented nor evaluated systematically, contributing to a disparity between the potential impact of novel approaches and the actual evidence of their effectiveness [9–11]. The fast-paced and highly pressured nature of healthcare delivery generally leaves limited time and resources for the comprehensive evaluation of newly introduced practices, particularly in environments marked by persistent resource constraints. Evaluation also has the potential to be seen as threatening to the intervention team, their colleagues and stakeholders [12]. The pressure to address urgent needs (such as waiting lists) can incentivise healthcare providers to prioritise rapid integration over rigorous evaluation [13]. The absence of incentives, both intrinsic and extrinsic, for thorough evaluation can perpetuate the cycle, preventing innovations from undergoing the scrutiny necessary to validate their impact. In addition, research capacity maybe limited; the intricacies of designing, conducting, and interpreting evaluations can be daunting, especially in an already demanding healthcare environment. This is particularly the case for innovations that encompass multiple elements, being implemented within a complex adaptive system [14]. Evaluations here call for something other than the prevailing linear, reductionist approaches, and require the expertise of several academic disciplines [15].

This lack of rigorous documentation and evaluation of health service innovations can hinder the broader understanding of what works best in what circumstances. Without an assessment of these innovations, their scalability, spread and sustainability remain uncertain, making it challenging to discern which strategies could yield the greatest benefits across different healthcare settings [16, 17]. The absence of formal documentation and evaluation processes also raises questions about equitable access to quality care. Innovations may lead to unintended consequences, particularly if variations in outcomes arise due to factors that were not adequately considered. Furthermore, the absence of clear documentation can limit the potential for shared learning and collaboration among healthcare providers [10]. Addressing this gap requires a concerted effort to integrate robust evaluation mechanisms into the fabric of cancer care innovations. By incorporating systematic documentation and rigorous evaluation from the outset, healthcare providers can foster a culture of continuous improvement, wherein innovations are refined and adapted based on evidence of their impact.

Cancer Research UK (CRUK) has been a key part of the concerted effort to build a body of evidence that supports healthcare commissioners and providers select the most impactful approaches. Building on their ‘ACE’ programme (2014–2019), which set out to Accelerate, Coordinate and Evaluate a range of innovative approaches being taken across the UK to improve cancer pathways, they have now launched the ‘Test Evidence Transition’ (TET) programme [18, 19]. This programme aims to accelerate the effective adoption of innovations, whilst working to reduce inequality in access to proven interventions. Through the provision of funding and by fostering a network and collaborative approach, TET will provide protected ‘testing’ spaces in which to explore and evaluate pathway innovations.

### The TET projects in Scotland

Two of the projects funded in the first wave of the TET programme are collaborations between two Scottish NHS Boards (NHS Fife and NHS Forth Valley), the National Centre for Sustainable Delivery (NHS Scotland) and multi-disciplinary academic teams at the University of Stirling. They both aim to optimise the diagnostic pathway for patients with suspected cancer. Whilst the cancer type (breast in Forth Valley, prostate in Fife) and the pathway changes are different in the two sites, the approach taken in the two projects is the same. Our overarching objectives are: (1) to support, monitor and evaluate improvements to cancer diagnostic pathways, and in doing so to move towards more efficient, effective, person-centred care; (2) to contribute towards the potential spread and adoption of the pathway improvements. The projects each last 18 months and run concurrently (from May 2023) with an overarching management team (with public contributors), stakeholder group and core research team (including social science, health services research, health economics, and qualitative and quantitative research expertise).

The aim of this paper is to describe and explain our research design and intended approach and activities. This is to support quality and transparency in research, to inform the scientific community and help coordinate research efforts, and to disseminate and discuss contemporary ideas with respect to study design.

## Methods

### Design and setting

The projects are set within two of Scotland’s 14 territorial Health Boards: NHS Forth Valley and NHS Fife. Both areas are in central Scotland, UK. The planned improvements were already conceived, based on previous analyses of data, feedback from and consultation with patients, and discussions amongst clinicians and managers. However, implementation had not begun prior to project inception. Both projects focus on improving cancer diagnosis pathways.

In Forth Valley, the improvement entails removing the need for a general practitioner appointment prior to referrals to the NHS breast assessment clinic. Patients calling their general practice reporting a breast lump will be assessed by a receptionist for eligibility for a rapid access breast clinic pathway, based on simple criteria to rule out potential breast abscess or breast-feeding problems. The decision to refer patients to the breast clinic will be forwarded to a team member with access to the referral system (i.e., SCI (Scottish Care Information) Gateway). Up to 49 general practices are anticipated to implement this new pathway.

In Fife, the improvement entails shifting key tasks and responsibilities in the prostate cancer diagnostic pathway from Urology Consultants to Advanced Clinical Nurse Specialists (ACNS), who will be supported by Patient Pathway Navigators (PPN). Eligible patients referred with suspected prostate cancer will attend a diagnostic clinic run by the ACNS and PPN, who will continue to see the patient through to decision to treat. Up to three nurse-led clinics per week are anticipated.

Since we are seeking to understand potential improvements within a complex system, we are adopting a hybrid effectiveness-implementation design that will assess both the efficiency of the new pathway and its implementation strategy, in support of rapid translation [20]. The evaluation will assess a range of implementation, service and clinical outcomes as determined by the Theory of Change (ToC) and seek to understand and/or explain what influences implementation outcomes such as acceptability, appropriateness, costs, feasibility and fidelity. Service and patient outcomes of particular interest will include access and equity, patient safety, clinical outcomes, resource utilisation, patient experience and timely diagnosis. To enable sufficient exploration of context and process, a naturalistic case study design will be used. This design is ideally suited to real-world, sustainable intervention development and evaluation where exposure to the intervention occurs in natural circumstances [21]. Where appropriate, outcomes will be assessed prior to and following the intervention. This design allows for in-depth exploration of the intervention, its implementation, and the context in which it is implemented. This can provide a rich understanding of the complexities of the intervention and help to identify factors that may influence its effectiveness and implementation. It can also help to identify changes and developments over the implementation period. Furthermore, the case study design allows for the collection and synthesis of data from multiple sources, helping to provide a comprehensive understanding of the intervention and its impact. Given the similarities in the two projects, there are also excellent opportunities for cross-fertilisation of ideas and an overarching synthesis of study findings.

### Patient and public involvement (PPI)

The projects are supported by a patient/public representatives’ group (n = 4) established in July 2023. We follow the UK Standards for Public Involvement to ensure the quality and consistency of how representatives are involved in the project [22]. Patient/public representatives were recruited through established contacts, charities (e.g., Breast Cancer Now) and the 1000 Elders Group at the University of Stirling. Interested members of the public contacted the researcher with a short paragraph about why they were interested in the projects, and subsequently met with the team’s PPI lead to discuss the opportunity further.

Patient/public representatives have a lived experience of breast (n = 2), prostate (n = 1), or another form of cancer (n = 1) and thus provide a lay perspective into the research. They contribute in two ways: first, they attend management group meetings; second, the research team meets with them as a PPI group to discuss various project aspects, as mentioned in the procedures and measures section below. We reimburse them for their time, and travel expenses are covered. Recognising the post-Covid-19 shift to hybrid working, meetings are a mixture of face-to-face and remote sessions [23]. Patient/public representatives receive ongoing support from the research team through email, and peer support in a WhatsApp group. They are invited to co-author external publications (such as this paper).

We have also conducted two small group discussions with current breast and prostate cancer patients in Forth Valley and Fife, to include perspectives of those who recently went through cancer pathways. We discussed their recent experiences of referral (for breast cancer) and from referral to diagnosis (for prostate cancer). Thereafter, we gave a brief overview of the project and gathered patients’ thoughts on how this could have changed their experience. The discussion contributed to the ToC for each project. These sessions were hosted by Maggie’s, a national charity providing free cancer support with local centres in Fife and Forth Valley. Patient/public representatives and the research team are involved in ongoing reflection of our work together to identify any issues or potential improvements. The impact of patient/public involvement in these projects will be reported using the GRIPP2 checklist [24].

### Procedures and measures

**Fig. 1:**
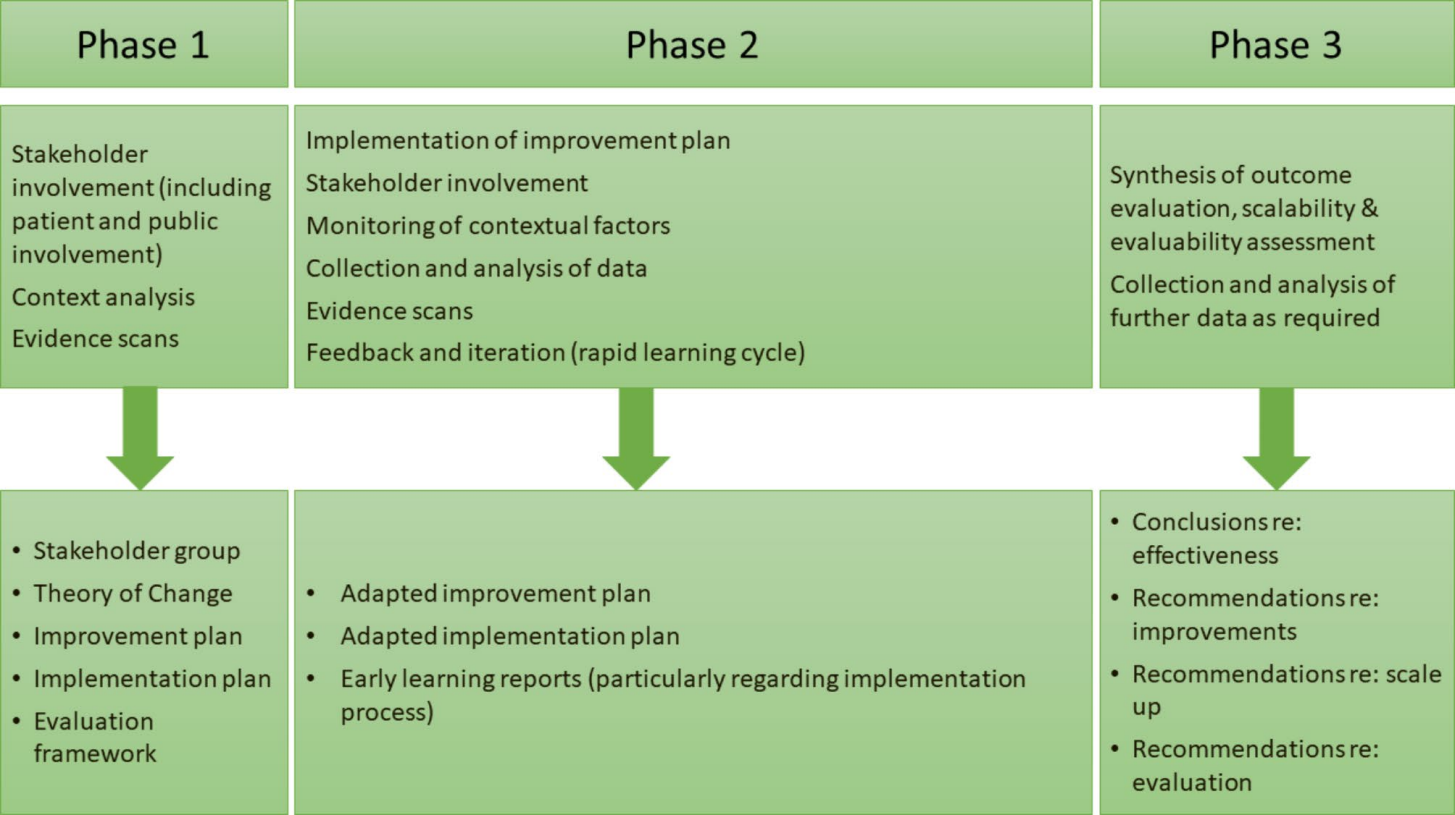
Summary of the three phases of the projects

The projects are divided into three interrelated phases: (1) the preparation phase, (2) the implementation and evaluation phase, and (3) the scalability assessment phase (see Fig. 1).

#### Phase 1

Lasting approximately three months, phase one involved preparatory activities that included establishing working relationships, identifying relevant stakeholders, consulting with members of the public, gathering background and contextual information, refining the improvement project and implementation plan, and finalising the research protocol. We gathered information to enable us to consider three related dimensions that fall within an Evaluability Assessment approach: evaluability of the projects ‘in principle’, as seen in the quality of the project design; evaluability ‘in practice’, as seen in the potential availability of data; and the utility and practicality of the evaluation, as seen in the institutional context. We used questions and criteria developed in previously conducted EAs, which proved to be valuable for informing the design and evaluation of new interventions [25, 26].

The team developed ‘swimlane’ process maps (cross-functional flowcharts) to analyse the current diagnostic processes, examine the groups or individuals that perform each step in the processes, and model future/improved processes in each site. Developing and discussing these process maps with different stakeholders and across the two projects led to improved communication within the teams and informed iteration of the improvement and implementation plans. We then developed ToC models for each project which described our desired changes, and how and why we expect these to happen. In each case, the process of model construction facilitated the development of our hypotheses, an assessment of the evidence-base underpinning these hypotheses, the articulation of key assumptions, and a discussion of their reasonableness and sensitivity to context. These ToC models formed the basis of discussions with public contributors and other stakeholders, who usefully challenged our theories and assumptions from different perspectives. They also formed the basis of our evaluation plan for phase two.

#### Phase 2

Phase two begins with the phased implementation of the new pathways, which will progress with learning and feedback from the evaluation. To evaluate implementation and outcomes, explore mechanisms and test assumptions within our ToC, we plan to conduct and analyse a range of data including routine quantitative data, patient survey data, patient interview data, staff survey (Forth Valley only) and interview data, and observational, documentary and other data such as self-reports/audits by the clinical team (see Table 1).

**Table 1:** Summary of measures and data sources

Data sources	When and How Many	Measures
Routine quantitative data	3 time points: - pre-pandemic (3-month period) (n = approximately 900 patients); - pre-implementation (3-month period) (n = approximately 1200 patients); - implementation (9-month period) (n = approximately 2700 patients)	• Patient demographics - Year of birth - Postcode of residence (for estimation of deprivation, rurality and proximity) - Gender - Ethnicity • Referral details • Time between referral and clinic appointment • Investigations • Diagnosis details - Date - Staging • Decision to treat date • Time between referral and decision to treat.
Patient survey data, using bespoke non-validated survey	Throughout implementation period. Anticipated size of sample: Fife – 540 Forth Valley – 1200	• Patient experience in relation to - Being referred - Costs of attending appointments - Information received and assessments conducted prior to clinic assessment - Attending the diagnostic clinic - Perceived quality of care within clinic • Patient demographics - Year of birth - Gender identity - Ethnic group - Postcode of residence (for deprivation estimation) - Registered GP practice
Patient interview data	Towards the end of implementation period. Anticipated size of sample: up to 15 in each site.	These in-depth semi-structured interviews are anticipated to follow up on specific aspects of patient experience and might be targeted towards further understanding experiences for people with specific characteristics.
Staff survey data (Forth Valley only)	At least halfway through the implementation period. Anticipated size of sample: 120	• Staff experience in relation to - Training needs for implementation of new pathway - Perceived challenges and benefits of the new pathway - Patients’ suitability for the new pathway • Costs to healthcare system - Time taken for key tasks/processes - Impact (if any) on existing alternative pathways - Impact (if any) on workloads • Staff characteristics - Job role - Place of work - Involvement in the pathway
Staff interview data	Month 6 and Month 12. Anticipated size of sample: up to 20 in each site from purposively selected staff	These in-depth semi-structured interviews are anticipated to follow up on specific aspects of staff experience and perceptions and will be particularly important for identifying/exploring any unintended consequences.
Project documentation, other observations and self-report/ audit by the implementation team.	Throughout phases 1 and 2	The constructs to be considered include: • Adoption – which consultants are involved, how are they brought on board, and what factors facilitate/hinder this process? • Implementation – how was the improvement plan delivered, including adjustments and adaptations? What implementation challenges were faced? What resources were required? • Sustainability – what is needed to sustain the improvement project? How should the new pathway be monitored/evaluated in the longer term? What modifications might be needed to sustain (or scale up) the improvement project?

##### Routine quantitative data

Routine quantitative data will be collated by the project managers within the NHS Boards, with the support of an information analyst. All personal identifying information will be removed, and the data will be transferred to the research team for analysis in accordance with our data sharing agreement and data management plan. Data will be extracted/collated for 3 time periods:

i. For a period of 3 months in a pre-pandemic period (in 2019), to analyse key measures in what might be considered a ‘business as usual’ environment.
ii. For a period of 3 months immediately prior to implementation, to analyse key measures immediately prior to the change in pathway (post-pandemic).
iii. For 9 months in phase 2, in order to analyse key measures following the change in pathway.

##### Patient survey data

All eligible patients (18 years or older and capable of consenting) referred to the relevant diagnostic clinic during a 9-month period within the implementation phase (approximate sample size 540 in Fife and 1200 in Forth Valley) will be asked to complete a short online questionnaire. The questionnaire was informed by work in phase one, and designed within the Jisc Online Surveys tool, with input from stakeholders and patient/public representatives. It will be optimised for completion on computer, tablet or mobile phone. It will be pilot tested with a sub-sample (N = ~ 20) and refined as necessary prior to use. Participants will be recruited in the clinic (by clinic staff/patient navigators) and encouraged to complete it within clinic (for Forth Valley) or at home (for Fife) within four weeks of attendance at the clinic. Two reminders will be sent (via phone, email or text message) to complete the survey within the four-week period. Patients will be offered alternative methods of completion, either on paper, or over the phone with a member of the research team. Informed consent will be via an ‘opt-in’ process, prior to survey completion. Survey respondents will be asked if they are interested in a potential future interview. If interested, they will be asked to provide name and contact details in a separate form.

##### Patient interview data

Depending on the need to follow up aspects of the survey findings, a small subset of up to 15 patients in each site may be invited to take part in a semi-structured telephone interview with an experienced researcher, lasting approximately 30 min. This will take place towards the end of the implementation period. Two options will be explored for identifying interview participants: (1) the study team will (randomly) select participants from those who expressed interest in participating when they completed the survey; (2) the study team will construct a purposive sampling frame, to be used by the clinical team to identify a sample of potential participants. Keeping these two options open gives us more scope to further explore qualitatively any issues arising from our quantitative data. Self-selected participants might have aspects of their experience they wish to share but may have certain biases as a sample. A purposive sampling frame would give us the option of finding out more about the experiences of particular demographics/sub-groups. Exclusion criteria will be those who are currently undergoing radical treatment, those who are unable to give informed consent, or those who have contraindications (e.g., symptoms or medical conditions) that are a reason for a person not to be included as a participant because it may be unreasonably difficult or distressing. Interview guides will be developed with input from stakeholders and patient/public representatives and will be informed by the analysis of the patient surveys. The interviews will be audio-recorded with permission, anonymised and transcribed.

##### Staff survey data (Forth Valley only)

Key staff in all participating general practices involved in the new pathway will be invited to complete an online questionnaire (at approximately month ten) to gather data related to the implementation process (approximate sample size 120). The questionnaire will be designed, developed, tested and refined in the same way as the patient questionnaire, with input from relevant stakeholders and patient/public representatives. The survey link will be sent to purposively selected staff by the project manager via NHS email, with opt-in informed consent. Up to two blanket reminder emails will be sent. Participants will not be asked for their name or any contact information. However, they will be asked to enter their job role and place of work. To maintain respondent confidentiality, the original dataset will be anonymised prior to analysis.

##### Staff interview data

Purposive samples of staff involved in implementing the improvement projects will be interviewed in approximately month 6 and month 12 (n = ~ 10 in NHS Forth Valley and n = ~ 20 in NHS Fife). A purposive sampling frame will be developed by the study team. Interview guides will be developed with input from stakeholders and patient/public representatives and will be informed by the analysis of other data. Interviews will be conducted with consenting participants by an experienced member of the research team either in person, by telephone, or via Microsoft Teams, depending on the participant’s preference. They will be recorded, anonymised and transcribed as for the patient interviews.

##### Project documentation

A range of other data such as meeting notes, action plans, team discussions, self-reports/audits by the implementation team, and observations will be collected to examine the implementation of the intervention and the proposed implementation for scale up. The collection of this data will be facilitated by the participatory implementation process and close working of all relevant stakeholders. Data will focus on assessing fidelity (in relation to the implementation plan) and adaptation, adoption and acceptability (particularly by different demographic groups and amongst different staff), delivery settings and workforce, implementation infrastructure, and sustainability.

##### Data analysis

All data sources will be analysed separately as one piece of a jigsaw, with each piece contributing to understanding of the whole phenomenon [27]. Qualitative data will be analysed thematically in NVivo 20, using Braun and Clarke’s reflexive approach [28]. Coding of data will be both inductive and deductive, based on our ToC, with analysis informing specific aspects of the evaluation framework and questions within the Interventional Scalability Assessment Tool (ISAT) [29]. Coding will be principally performed by the research fellows (MMc and PT) who are experienced in qualitative analysis, with samples of the data also being coded by two other members of the research team to explore coder consistency and to highlight issues for whole team discussion. Where appropriate, our PPI members will be asked to inform aspects of analysis and interpretation through feedback and discussion.

Quantitative data will be analysed within IBM SPSS. Where appropriate, specific outcome variables will be compared at multiple time points before and after the intervention is implemented using interrupted time series analysis, to determine whether the change in pathway has an effect that is statistically significantly greater than the underlying trend (e.g., to examine the trends in time to cancer diagnosis for people with possible symptoms of cancer). This is a pragmatic choice of method that will ensure a limited impact of selection bias and confounding due to population differences. However, it is limited in that it will not control for confounding as a result of other interventions or events occurring at the same time as the intervention. This will be mitigated by analysing data both before and after one significant event (the onset of the Covid-19 pandemic), and by working closely with stakeholders to ensure a good understanding of (historical) context.

Economic analyses will be performed to identify the nature of the impact (if any) on the resource use of the two patient groups (pre and post intervention). The work is intended to be exploratory and will provide a sound basis for future cost effectiveness analyses in this area. Three analyses are proposed: a comparison of costs and outcomes of pre- and post-implementation pathways using decision analytical modelling [30]; the analysis of demographic data (including deprivation indicators) alongside clinical outcome indicators of interest to identify any relevant relationship and impact of the intervention on health inequalities, and; an exploratory analysis of the impact the intervention has had on the socioeconomic costs of the patients on each pathway, using data from the patient surveys.

Initial data analysis will be ongoing throughout phase two, to enable findings to be fed back to the implementation team for ongoing improvement.

#### Phase 3

Data collection and information sources in phase two are geared towards enabling us to answer relevant questions posed in the ISAT. This tool was developed through a review of the implementation science literature and several rounds of input from implementation researchers, policy makers and practitioners actively involved in program management and/or the scaling up of health interventions and programs [29]. We will use it within phase three to assist in assessing the scalability of the improvement projects, as well as to identify and assess contextual factors that might help or hinder scale up.

All data from phase two will be collated by the University research team, anonymised and organised according to the ToC and to the domains within the ISAT. When the first stage of analysis is complete, data will be reduced to a series of thematic statements for each data source making sure we do not lose too much detail [31]. We will then conduct pattern-matching across the data, seek rival explanations, link data to propositions (generated by our ToC), and build explanations. To support this, a number of analytical questions will be developed by the project team and stakeholders to underpin our aims and aid consistency of analytical focus. Organisational, behavioural and implementation theories will be employed, alongside PPI input, to inform interpretation of data.

### Ethics

These projects are examples of service development/improvement, which seek to find out what improvement can be achieved within a specific service. Since they are designed to produce potentially transferable findings, in that the context and findings will be described and defined so that the conclusions can be applied or transferred to other settings, both projects are considered research by the NHS Health Research Authority. Since the research involves prospective collection of information from users of NHS services, where research use is intended at the time of collection, we sought NHS Research Ethics Committee review. Ethics approval was granted for both projects prior to phase two (NHS Fife, 23/SC/0252; NHS Forth Valley, 23/EE/0168).

## Discussion

This paper describes the approach taken within two projects funded as part of the CRUK ‘Test Evidence Transition’ programme, which blend implementation research and embedded case study design. Implementation science offers a structured framework for introducing, documenting, evaluating, and disseminating innovations within real-world healthcare settings. It provides a systematic approach to understanding not only whether an innovation is effective but also how it can be integrated successfully into the existing healthcare landscape [32]. Consistent with an implementation science approach, our evaluations seek to answer critical questions such as how innovations can be adapted to suit different contexts, how barriers to adoption can be mitigated, and how the long-term sustainability of these innovations can be ensured.

Amid the complexity of cancer care innovations and the constraints of limited resources, the need for pragmatic, participatory evaluation designs is increasingly apparent. These designs prioritise not only the rigorous assessment of the effectiveness of innovations but also the active engagement of stakeholders throughout the evaluation process. By involving healthcare providers, patients, policymakers, and other relevant parties, such research can harness the collective wisdom and insights needed to understand how innovations function within the real-world context of healthcare delivery [33, 34]. This collaborative approach not only enhances the credibility, relevance and acceptability of the evaluation but also helps to strengthen research capacity amongst healthcare professionals.

Rather than seeking to create a controlled environment, our research design acknowledges the inherent complexities of delivering care and aims to capture the multifaceted factors that influence innovation outcomes. This approach provides a more holistic understanding of how an innovation interacts with the broader healthcare ecosystem and enables researchers to identify barriers, facilitators, and unanticipated consequences that can impact scalability, sustainability and spread. By opting for a pragmatic, participatory evaluation research design, we hope to inform strategies for scaling up successful innovations while addressing challenges in a targeted manner. In doing so, such research helps to bridge the gap between innovation and practice.

## Data Availability

Not applicable.

## List of abbreviations

ACE programme: Accelerate, Coordinate, Evaluate programme
ACNS: Advanced Clinical Nurse Specialist
CRUK: Cancer Research UK
GP: General Practice
ISAT: Intervention Scalability Assessment Tool
NHS: National Health Service
PPI: Patient and Public Involvement
PPN: Patient Pathway Navigator
SCI: Scottish Care Information
TET programme: Test, Evidence, Transition programme
ToC: Theory of Change
